# Effects of Laurocerasus Officinalis Roem (Cherry Laurel) on Cognitive Function and Neurobiochemical Pathways in a Streptozotocin-Induced Nontransgenic Alzheimer’s Disease Model

**DOI:** 10.3390/nu18050867

**Published:** 2026-03-08

**Authors:** Fulya Ozsoy, Karolin Yanar, Ugurcan Sayili, Pinar Atukeren, Hafize Uzun

**Affiliations:** 1 Department of Biochemistry, Cerrahpaşa Faculty of Medicine, Istanbul University-Cerrahpasa, 34098 Istanbul, Turkey; ozsoyfulya@gmail.com (F.O.); yanarkarolin@gmail.com (K.Y.); p_atukeren@yahoo.com (P.A.); 2 Medical Laboratory Techniques Programme, Vacotinial School and Isik University, 34980 İstanbul, Turkey; 3 Department of Public Health, Cerrahpasa Faculty of Medicine, Istanbul University-Cerrahpasa, 34098 Istanbul, Turkey; ugurcan.sayili@iuc.edu.tr; 4 Department of Medical Biochemistry, Faculty of Medicine, Istanbul Atlas University, 34303 Istanbul, Turkey

**Keywords:** Alzheimer’s disease, type 2 diabetes, cherry laurel, Moris Water Maze, GSK3-β

## Abstract

**Background:** This study investigated the effects of *Laurocerasus officinalis* Roem (cherry laurel; CL), a traditionally consumed fruit, on cognitive performance and selected neurobiochemical and metabolic pathways in a nontransgenic streptozotocin (STZ)-induced Alzheimer’s disease (i.c.v. STZ) model and an STZ-induced type 2 diabetes mellitus (T2DM; i.p. STZ) model. **Method:** Fifty-seven adult male Sprague–Dawley rats were allocated to control, T2DM, and Alzheimer (ALZ) model groups, with subgroup interventions including CL supplementation and, in the T2DM model, metformin as a comparator. Spatial learning and memory were assessed using the Morris Water Maze. Serum and brain tissue levels of GSK3-β, glutathione (GSH), interleukin-1 (IL-1), GLUT4, GLP-1, β-amyloid (Aβ), and acetylcholinesterase (AChE) were quantified. **Results:** Serum GSK3-β levels did not differ significantly between groups, whereas brain tissue GSK3-β showed significant between-group differences. CL increased GSH levels in both models, with significant elevations in serum and brain tissue GSH in the ALZ model following CL administration; in the T2DM model, GSH increased after both CL and metformin. In the ALZ model, CL was associated with decreased serum Aβ and AChE levels and improved Morris Water Maze performance, reflected by reduced escape latencies. **Conclusions:** CL supplementation was associated with antioxidant enhancement and modulation of amyloid- and cholinergic-related measures, alongside improved spatial learning performance in the STZ-induced nontransgenic ALZ model. In addition, CL reduced blood glucose in the T2DM model. Given the likely contribution of fruit phytochemicals (including total phenolics), further studies are warranted to better define the bioactive composition and mechanisms underlying these effects.

## 1. Introduction

Alzheimer’s disease (AD) is the most common cause of dementia and is characterized by progressive cognitive decline. Increasing evidence supports substantial mechanistic overlap between AD and metabolic disorders, particularly type 2 diabetes mellitus (T2DM) [1]. In this context, the term “type 3 diabetes mellitus (T3DM)” has been used to describe brain insulin resistance and impaired cerebral glucose utilization, which may contribute to synaptic dysfunction, neuroinflammation, and cognitive impairment in sporadic AD. Accordingly, experimental models that reproduce metabolic/insulin-related disturbances in the brain provide a relevant framework for studying AD–T2DM convergence and for testing nutraceutical interventions [2].

A central signaling node in this overlap is glycogen synthase kinase 3-β (GSK3-β), which is implicated in neurodegeneration and metabolic dysregulation [3]. In parallel, oxidative stress and neuroinflammation contribute to disease progression: glutathione (GSH) is a key endogenous antioxidant that tends to be reduced in chronic diabetes and AD, while interleukin-1β (IL-1β) is a major proinflammatory mediator implicated in both metabolic inflammation and AD-related neuroinflammatory cascades [4,5,6,7]. Moreover, impaired glucose handling may directly affect cognition; glucose transporter 4 (GLUT4) and incretin-related signaling (GLP-1) are linked to insulin sensitivity and neuronal energy balance, and GLP-1-related pathways have been associated with improved cognitive outcomes in experimental AD settings [8]. Finally, amyloid-β (Aβ) dynamics and cholinergic dysfunction are hallmarks of AD; acetylcholinesterase (AChE) contributes to cholinergic deficit and may facilitate Aβ-related pathology [9].

Glucagon-like peptide-1 (GLP-1) is an incretin family peptide hormone containing 30 amino acids. In experimental Alzheimer’s models, the use of GLP-1 analogues has been shown to improve cognitive functions and reduce amyloid plaque accumulation and tau hyperphosphorylation. It has been demonstrated that GLP-1 analogues are especially involved in the inhibition of − 3β which plays a role in tau phosphorylation by activation of AKT signalling system in the cell [10]. Acetylcholinesterase enzyme (AChE) is a specific esterase belonging to the carboxylesterase enzyme family. AChE may play an important role in the pathogenesis of AD by promoting the formation of β-amyloid plaques in the cerebral cortex [11].

*Laurocerasus officinalis* Roem. (syn. *Prunus laurocerasus* L.), commonly known as cherry laurel (CL) or “taflan,” belongs to the Rosaceae family. It is an evergreen shrub or small tree native to the Black Sea region of Northern Türkiye, the Caucasus, and the Balkans. The plant is characterized by its coriaceous (leathery), oblong leaves and small, white flowers arranged in racemes. The fruits are drupes that turn dark purple or black upon ripening and are widely consumed fresh, dried, or as traditional preserves (jam/molasses) [12]. Phytochemical analysis reveals that fruit is a potent source of bioactive compounds, particularly anthocyanins (such as cyanidin-3-glucoside), phenolic acids (chlorogenic acid), and flavonoids (quercetin derivatives). These components are primarily responsible for their high antioxidant capacity and their ability to modulate metabolic and neuroprotective pathways [13,14,15,16]. According to the Turkish National Food Composition Database (TürKomp) [17], cherry laurel fruit contains approximately 75 kcal energy per 100 g, with 79.85 g water, 14.72 g carbohydrates (including 5.09 g glucose and 6.36 g fructose), 2.81 g dietary fiber, and 1.40 g protein. Furthermore, it contains essential micronutrients, including vitamin C (2.3 mg), vitamin A (2 RE), and iron (0.55 mg), which establish it as a nutritionally and metabolically significant fruit matrix [17].

Beyond its traditional use as an antitussive and diuretic agent for gastrointestinal and respiratory disorders, *L. officinalis* is recognized for its potent immune-modulatory and antioxidant properties [18,19]. Recent studies highlight its therapeutic potential in disease prevention, primarily attributed to its high concentration of flavonoids, phenolic compounds, and essential fatty acids [20,21].

Prior work has suggested neuroprotective potential in experimental settings, and additional studies have reported favorable biochemical/antioxidant profiles in diabetic models, providing a rationale to explore its effects in the AD–T2DM axis. Importantly, we focused on fruit because it represents the commonly consumed edible form with direct translational relevance and because our phytochemical characterization indicates a rich phenolic content likely contributing to biological activity [14].

We hypothesized that CL fruit supplementation would improve learning/memory performance in STZ-induced nontransgenic AD and T2DM models by modulating a mechanistic network involving (i) antioxidant defense (GSH), (ii) inflammatory signaling (IL-1β), and (iii) metabolic/insulin-linked pathways (GLUT4/GLP-1), with downstream effects on GSK3-β and AD-related readouts (Aβ and AChE). Therefore, we evaluated cognitive function (Morris Water Maze) and quantified these biomarkers in both serum and brain tissue to test tissue-specific effects and mechanistic consistency across the two models.

## 2. Materials and Methods

In this study, approval was obtained from Bezmialem Vakıf University Animal Experiments Local Ethics Committee with the decision numbered 2017/196 on 20 July 2017. All procedures were conducted in accordance with the NIH Guide for the Care and Use of Laboratory Animals. In the study, 57 5–6 months old adult male Sprague–Dawley rats with an average weight of 450 ± 50 g were obtained from the same laboratory.

The number of animals per group was calculated by the “One-Way ANOVA Power Analysis” method. With this analysis, the number of animals was determined as 5 for control groups and 9 for study groups. However, considering the possibility of animal losses in STZ-induced experimental T2DM and Alzheimer’s model, the number of animals per group was increased by one in the study groups.

The rats in the control group were randomly divided into two cages with 5 rats in each cage, and the rats in the study group were divided into two cages with four and five rats for each group. During the experiments, the rats were kept under standard housing conditions (room temperature 21 ± 2 °C and humidity 60–65%) with ad libitum feed and water intake in a 12 h light–12 h dark cycle. 

### 2.1. Cherry Laurel (CL) Preparation and Administration

CL fruits were obtained from a certified local supplier. Botanical identification was based on supplier documentation and macroscopic characteristics consistent with published species descriptions. No independent taxonomic authentication was performed. The present study did not quantify individual daily intakes of specific bioactive compounds (e.g., total polyphenols, anthocyanins, carotenoids or terpenoids). However, previous phytochemical analyses have consistently reported high phenolic and anthocyanin content in CL fruit [13,14,15,16,19], supporting the biological plausibility of antioxidant-mediated effects.

To minimize potential exposure to cyanogenic glycosides, which are reported to be concentrated particularly in the kernels/seeds (and variably present in other parts depending on ripening), all seeds were removed and only the edible pulp was used. The pulp was weighed and homogenized under cold conditions to obtain standardized preparation.

The selected concentration (0.37 g/mL) was based on previously published in vivo studies evaluating the antioxidant and antidiabetic effects of *Laurocerasus officinalis* fruit preparations in STZ-induced rat models [22,23,24]. These studies demonstrated biochemical efficacy without observable toxicity during short-term intervention periods. The present study aimed to evaluate the biological effect of the whole fruit matrix rather than isolated compounds; therefore, the administered preparation reflects a nutritionally relevant whole fruit homogenate.

The homogenate was prepared as a 0.37 g/mL (*w*/*v*) suspension in distilled water, aliquoted, and stored at −20 °C until use. Immediately before administration, an aliquot was thawed and vortexed to ensure homogeneity. The CL fruits were administered via oral gavage once daily each morning for 21 days to the relevant groups (control + CL, T2DM + CL, and ALZ + CL). The gavage volume was adjusted according to body weight and maintained within standard limits for oral gavage in rats. Oral dosing schedules were consistent with previous rat studies using CL fruit preparations. CL was orally applied by gavage at the level of 10 mL/kg, b.w. [b.w.: body weight] for 21 days [25].

No acute toxicity, behavioral distress, or metabolic deterioration was observed during the 21-day CL administration period.

### 2.2. Intracerebroventricular STZ INJECTION

For intracerebroventricular STZ injection, the artificial cerebrospinal fluid (aCSF) to be used to dissolve STZ was prepared beforehand. To prevent the aCSF from deteriorating, it was prepared in two separate falconries, solution A and solution B, and stored at 4 °C. STZ was administered intravenously to the animals at a dose of 3 mg/kg in 20 μL of aCSF in two doses, one day apart.

Group 1 (Control group, n = 5): Rats were injected bilaterally with 20 µL of artificial cerebrospinal fluid intracerebroventricularly (i.c.v) on the first and third days. The weights and tail blood glucose levels of the rats were measured three days before and after i.c.v. injection of artificial cerebrospinal fluid on the first day and every three days during the experiment. 

Group 2 (Control + Cherry Laurel (CL), group, n = 5): Rats were injected bilaterally with 20 µL of artificial cerebrospinal fluid i.c.v. on the first and third days. The weights and tail blood glucose levels of the rats were measured three days before and after i.c.v. injection of artificial cerebrospinal fluid on the first day and every three days during the experiment. The rats were given 0.37 g/mL CL by gavage every morning. 

Group 3 (T2DM group, n = 8): This is the experimental model in which T2DM model was created. The 65 mg/kg intraperitoneal STZ dose was chosen in accordance with established protocols for inducing experimental diabetes in rats [23,24]. The T2DM model was created by administering 65 mg/kg STZ (STZ, SIGMAS0130) intraperitoneally (i.p. on the first day. Weights and tail blood glucose levels were measured before and three days after STZ administration on the first day and every three days during the experiment. Rats with glucose levels above 200 mg/dL in tail blood samples were considered to be a T2DM model. 

Group 4 (T2DM + CL, group, n = 8): This is the experimental model in which the T2DM model was created and CL was given. The T2DM model was created by administering 65 mg/kg STZ i.p. on the first day. Weights and tail blood glucose levels were measured before and three days after STZ administration on the first day and every three days during the experiment. It was accepted that T2DM model occurred in rats with glucose levels above 200 mg/dL in tail blood samples. The rats were given 0.37 g/mL CL by gavage every morning for 21 days. 

Group 5 (T2DM + Oral Antidiabetic (OA) group, n = 7): This is the experimental model in which the T2DM model was created, and oral antidiabetics were given. The T2DM model was created by giving 65 mg/kg STZ i.p. on the first day. Weights and tail blood glucose levels were measured before and three days after STZ administration on the first day and every three days during the experiment. Rats with glucose levels above 200 mg/dL in tail blood samples were considered to be a T2DM model. Rats were given 300 mg/kg/day metformin by gavage for 21 days. 

Group 6 (Alzheimer (ALZ) group, n = 9): This is the experimental group in which the Alzheimer’s model was created. The 3 mg/kg intracerebroventricular STZ protocol was applied according to previously validated nontransgenic sporadic AD models [26,27]. On the first and third days, 3 mg/kg STZ was dissolved in artificial cerebrospinal fluid and injected into each ventricle with a Hamilton injector with 10 µL, totaling 20 µL per day. Weights and tail blood glucose levels were measured before, three days after and every three days during the experiment. 

Group 7 (ALZ + CL group, n = 8): This is the experimental group in which the Alzheimer’s model was created, and CL was given. STZ (3 mg/kg) was prepared in artificial cerebrospinal fluid and injected into each ventricle (10 µL/ventricle) via a Hamilton syringe on the first and third days, resulting in a total daily dose of 20 µL. Weights and tail blood glucose levels were measured before and three days after STZ administration on the first day and every three days during the experiment. Rats were given 0.37 g/mL CL by gavage every morning for 21 days. 

Experimental concentrations and model protocols were selected to ensure methodological consistency with previously validated STZ-based metabolic and neurodegenerative models. No acute toxicity or behavioral distress was observed during CL administration at the selected dose.

The rats in each group were subjected to the Morris Water Maze (MWM) test for 5 days starting from the 14th day. The rats lived for 21 days and were sacrificed on the 22nd day.

The experimental groups, induction protocols, and treatment regimens are summarized in Table 1

### 2.3. Obtaining Blood and Tissue Samples from Animals

At the end of the experimental period, Na-pentothal (35 mg/kg) was administered i.p. and anesthesia was performed and intracardiac blood was taken and sacrificed for the study of biochemistry parameters. Brain tissues obtained from rats were stored at −80 °C until biochemical analyses were performed. Intracardiac blood samples taken from rats were taken into tubes with EDTA. Plasma samples were obtained by centrifugation at 1000× *g* for 15 min in a refrigerated centrifuge device and the samples were divided into Eppendorf centrifuges and stored at −80 °C until biochemical analyses were performed. 

### 2.4. Tissue Homogenization

As hemolyzed blood in the tissues taken from rats may affect the results, the tissues were washed in cold phosphate buffer (0.01 M pH = 7.4) at the time of collection. Tissue pieces were weighed wet and disintegrated into small pieces using a glass homogenizer in phosphate buffer at +4 °C to prepare 10% (*w*/*v*) homogenates. The prepared homogenates were centrifuged at 5000× *g* for 5 min with a cooled Eppendorf centrifuge for biochemical parameters and the studies were performed with the samples taken from the supernatants. Supernatants were stored at −80 °C until the time of the study.

### 2.5. Morris Water Maze (MWM) Test

Spatial learning and memory were assessed using a circular Morris Water Maze (MWM) tank (150 cm diameter × 60 cm height). The tank was filled with water opacified with non-toxic food coloring and maintained at a constant temperature of 23 °C via an automatic heater. The maze was divided into four equal quadrants, with a hidden platform (11 × 11 cm) submerged 2 cm below the water surface in a randomly selected but fixed quadrant. Extramaze visual cues were positioned around the test room to facilitate spatial navigation.

For four consecutive days, rats underwent four trials per day, being released into the water facing the pool wall from a different starting location in each trial. All behavioral parameters, including swimming speed, distance, trajectory, and escape latency, were automatically recorded using EthoVision XT11 software (Noldus Information Technology, Wageningen, The Netherlands). If a rat failed to locate the platform within 60 s, it was manually guided to it by the researcher, allowed to remain there for 10 s, and then returned to its home cage.

**Figure 1:**
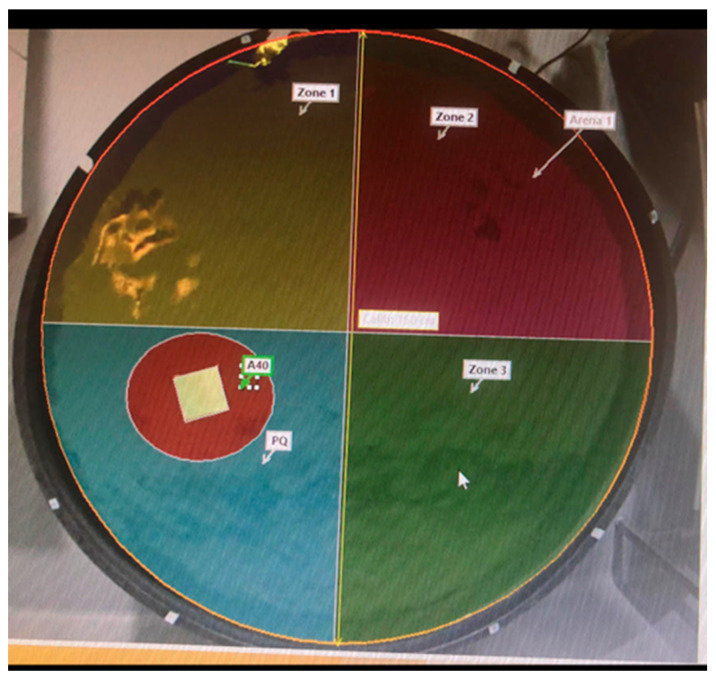
Morris Water Maze Tank.

On the fifth day, a probe trial was conducted by removing the platform. The rats were allowed to swim freely for 60 s; the distance traveled and the percentage of time spent within the target quadrant and a 40 cm diameter zone around the former platform location were measured. The experimental setup is illustrated in Figure 1.

### 2.6. Intracerebroventricular (i.c.v) Injection with Streptozotocin (STZ)

Animals were anesthetized intraperitoneally with a mixture of xylazine and ketamine hydrochloride (3 mg/kg and 75 mg/kg, respectively) prior to i.c.v. injection. The scalps were shaved, cleaned and cut to expose the skull. The heads of the rats were placed in a stereotaxic frame, and a midline sagittal incision was made in the scalp. Holes were made in the skull over the lateral ventricles using coordinates 0.8 mm posterior to the bregma, 1.5 mm lateral to the sagittal suture and 3.5 mm below the skull surface. STZ (3 mg/kg) was dissolved in artificial cerebrospinal fluid (3 mM KCl, 140 mM NaCl; 1.2 mM CaCl_2_, 1 mM MgCl_2_; 0.3 mM NaH_2_P0_4_, 1 mM Na_2_HP0_4_, 3 mM D-glucose, pH = 7.4). On the first and third days, a volume of 10 µL was injected slowly into each lateral ventricle with the same coordinates using a Hamilton injector. This solution was prepared immediately before injection.

### 2.7. Intraperitoneal (i.p.) Injection with Streptozotocin

To induce the T2DM model, streptozotocin (STZ; 65 mg/kg) was freshly dissolved in a 0.1 M citrate buffer (pH = 4.5), prepared using sodium citrate dihydrate (C_6_H_5_Na_3_O_7._ 2H_2_O) and citric acid (C_6_H_8_O_7_). The solution was administered via a single intraperitoneal (i.p.) injection at a volume of 2 mL per rat.

### 2.8. Analysis of Serum Biochemical Tests

Serum glucose, urea, creatinine, alanine transaminase (ALT), aspartate transaminase (AST), cholesterol, triglyceride (TG), high-density lipoprotein (HDL), low-density lipoprotein (LDL) levels were measured by Roche Cobas c702 (Mannheim, Germany).

Serum and brain tissue GSK3-β, GSH, IL-1β, GLUT4, Aβ, GLP-1, AChE levels were measured by the ELISA method (Rat ELISA Kits, Abbkine, China; Intra-Assay: CV (%) < 8% and Inter-Assay: CV (%) < 9%).

Protein content in brain tissues was determined by the bicinchoninic acid (BCA) method.

**Figure 2:**
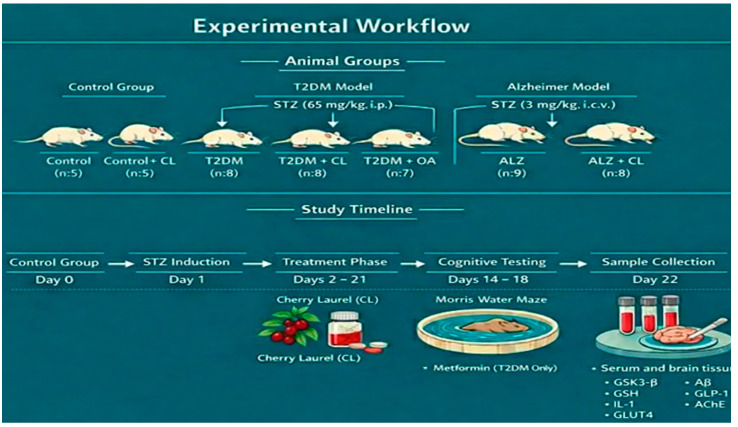
Experimental workflow of the study.

The overall experimental design, including group allocation, model induction, treatment schedule, behavioral testing, and sample collection, is summarized schematically in Figure 2 ((A) animal group allocation and model induction protocols, including intraperitoneal (i.p.) STZ administration for T2DM (65 mg/kg) and intracerebroventricular (i.c.v.) STZ administration for the nontransgenic Alzheimer model (3 mg/kg), (B) study timeline showing induction, treatment phase (cherry laurel or metformin), Morris Water Maze testing, and serum/brain tissue collection for biochemical analyses).

### 2.9. Statistical Analysis

The IBM SPSS (The Statistical Package for the Social Sciences) version 21.0 package program (USA) was used for data evaluation and analysis. The normal distribution of continuous variables was evaluated by a Shapiro–Wilk test. Data that met the normal distribution conditions were expressed as mean ± std; data that did not meet the normal distribution conditions were expressed as median (minimum-maximum). A one-way ANOVA test was used to compare normally distributed continuous variables of more than two independent groups. When a difference was found between groups by one-way ANOVA test, the post hoc Tukey method was used to determine the groups from which the difference originated. The Kruskal–Wallis test was used to compare continuous variables of more than two independent groups that were not normally distributed. When the Kruskal–Wallis test revealed a difference between groups, pairwise comparisons were made with the Bonferroni correction to determine the groups from which the difference originated. For statistical significance, *p* < 0.05 was accepted.

## 3. Results

Baseline serum glucose values did not differ significantly among the groups (*p* = 0.05). Following diabetes induction, intergroup differences became pronounced (*p* < 0.001). On the diabetes day, serum glucose levels in the T2DM (447 ± 53 mg/dL), T2DM + CL (447 ± 37 mg/dL), and T2DM + OA (246 ± 56 mg/dL) groups were significantly higher compared to the control (111 ± 12 mg/dL), control + CL (120 ± 8 mg/dL), ALZ (112 ± 9 mg/dL), and ALZ + CL (123 ± 2 mg/dL) groups. This pattern persisted throughout the experiment. On Day 7, glucose levels in the T2DM, T2DM + CL, and T2DM + OA groups were 455 ± 63, 367 ± 44, and 197 ± 67 mg/dL, respectively, while all remaining groups remained below 127 mg/dL (*p* < 0.001). On Day 14, the T2DM group reached the highest recorded value of 581 ± 41 mg/dL; T2DM + CL and T2DM + OA groups showed 272 ± 33 and 148 ± 19 mg/dL, respectively, whereas the control, control + CL, ALZ, and ALZ + CL groups remained within the normal range (109 ± 7, 115 ± 6, 100 ± 2, and 114 ± 3 mg/dL, respectively; *p* < 0.001). On Day 21, a similar pattern was observed, with the T2DM group maintaining 581 ± 23 mg/dL, T2DM + CL at 217 ± 33 mg/dL, and T2DM + OA at 148 ± 19 mg/dL, while the other groups ranged between 107 and 118 mg/dL (*p* < 0.001).

Baseline body weights did not differ significantly among the groups on Day 0 (*p* < 0.001). The control group had the lowest initial weight (259.6 ± 27.46 g), followed by control + CL (294.2 ± 20.1 g), while the remaining groups exhibited higher weights ranging from 322.67 ± 31.5 g (ALZ) to 378 ± 47.16 g (T2DM). Following diabetes induction, intergroup differences remained statistically significant (*p* = 0.002). On the diabetes day, the T2DM (345 ± 47.9 g), T2DM + CL (316.25 ± 33.97 g), T2DM + OA (317 ± 27.45 g), and ALZ + CL (319.63 ± 49.87 g) groups showed significantly higher weights compared to the control group (244 ± 28.18 g). On days 7 and 14, no statistically significant differences were observed among the groups (*p* = 0.061 and *p* = 0.065, respectively). Body weights ranged between 259 ± 31.84 g and 325.88 ± 54.25 g on Day 7 and between 275 ± 21.21 g and 326.75 ± 52.62 g on Day 14. On Day 21, intergroup differences regained statistical significance (*p* = 0.021). The ALZ (322.11 ± 29.76 g) and ALZ + CL (331.63 ± 50.9 g) groups maintained the highest weights, while the T2DM + OA (271 ± 26.97 g), T2DM + CL (275.25 ± 55.04 g), and T2DM (281.75 ± 46.89 g) groups exhibited the lowest values. The control and control + CL groups showed intermediate weights of 282.4 ± 28.18 g and 316.2 ± 28.87 g, respectively.

**Figure 3:**
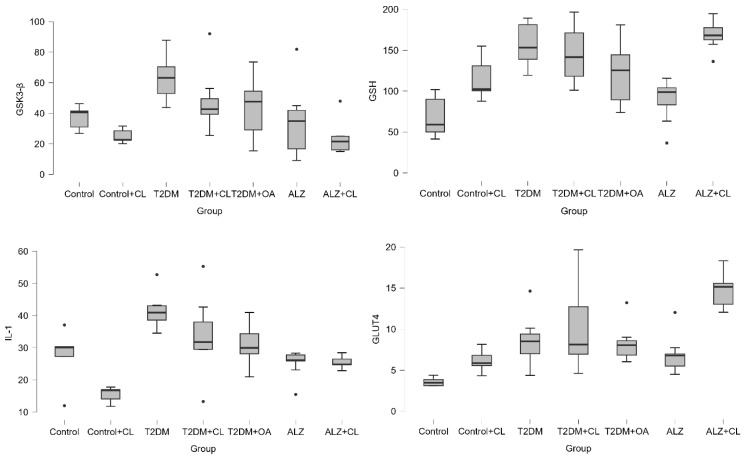

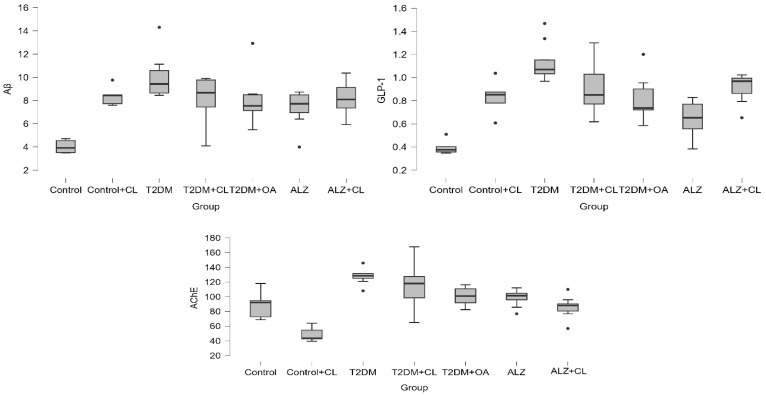
Brain Tissue Neurobiochemical Biomarker Levels Across Experimental Groups.

**Figure 4:**
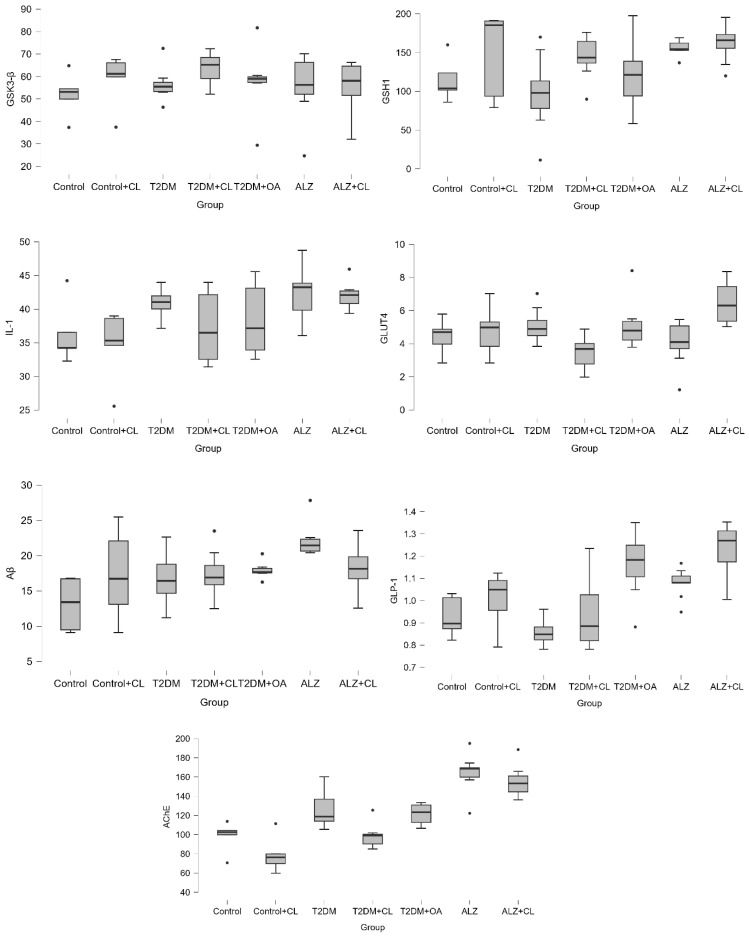
Serum Neurobiochemical Biomarker Levels Across Experimental Groups.

Brain tissue levels of GSK3-β, GSH, IL-1, GLUT4, β-Amyloid, GLP-1, and AChE are presented in Table 2 and Figure 3, while their respective serum levels are shown in Table 3 and Figure 4. General serum biochemical parameters, including renal and hepatic markers, as well as lipid profiles, are summarized in Table 4.

The spatial learning and memory performance evaluated by the Morris Water Maze (MWM) test, represented by swimming times (s), are provided in Table 5. On Day 1, statistical analysis of swimming times revealed a statistically significant difference between the experimental groups (*p* = 0.023).

## 4. Discussion

CL (*Laurocerasus officinalis*) fruit is known to contain a variety of bioactive compounds, including phenolic acids, flavonoids, anthocyanins, and other antioxidant constituents. In this study, CL supplementation was associated with improved behavioral performance in the Morris Water Maze, particularly in the STZ-induced nontransgenic ALZ model, alongside consistent biochemical changes across serum and brain tissue. Specifically, CL increased GSH levels (notably in both serum and brain in the ALZ + CL group), reduced serum Aβ and AChE levels in the ALZ model, and lowered blood glucose in the T2DM model. In addition, while serum GSK3-β levels did not differ significantly between groups, brain tissue GSK3-β showed significant between-group differences, indicating tissue-specific regulation. Together, these findings provide an integrated behavioral and biochemical profile of CL effects in experimental T2DM and STZ-induced sporadic Alzheimer models.

Although cherry laurel fruit contains approximately 14–15 g carbohydrates per 100 g [17], gavage volume was weight-adjusted and standardized across experimental groups. Importantly, CL administration did not increase serum glucose levels in non-diabetic groups, and glucose levels decreased in diabetic groups receiving CL. These findings suggest that the net metabolic effect observed in the present study was not attributable to simple sugar intake but rather to the bioactive composition of the fruit matrix. Blood glucose levels were significantly elevated in the T2DM group and were reduced after CL or metformin treatment. Across diabetic groups, glucose levels remained higher than in the control and ALZ groups. Although CL lowered blood glucose, metformin produced a greater reduction, consistent with its established glucose-lowering actions in STZ-based diabetic models (e.g., improved insulin sensitivity and reduced hepatic glucose output). A previous study comparing CL extract with glibenclamide also reported improved glycemic control and higher insulin levels in the CL-treated group [22,28]. These findings indicate that CL may contribute to glycemic improvement in the T2DM model; however, further studies are needed to define its active constituents and dose–response effects.

This study evaluated GSK3-β in both serum and brain tissue to address potential tissue-specific regulation. No significant differences were observed in serum GSK3-β levels among groups, whereas brain tissue GSK3-β levels were significantly elevated in the T2DM group compared to control, ALZ, and ALZ + CL groups. CL administration was associated with lower brain GSK3-β levels in the disease models. Taking together, these results suggest that GSK3-β alterations in this setting are primarily tissue-specific. Prior studies have reported that metformin can modulate GSK3-β expression/activity in experimental neurodegeneration and STZ-based sporadic AD models [26,27,29], supporting the relevance of this pathway in metabolic–neurodegenerative overlap.

Decreased brain glutathione (GSH) has been associated with neurodegenerative disorders and has been assessed in patients using ^1^H-MRS [30]. In the present study, CL increased GSH in both models, with significantly higher brain and serum GSH levels in the ALZ + CL group compared with relevant comparator groups and increased serum GSH following CL intake in both ALZ and T2DM models. In the T2DM model, GSH also increased after metformin. While methodological differences limit direct comparison across studies, our findings are consistent with reports that CL preparations can improve oxidative stress markers, including GSH, in STZ-induced diabetic models [23,24].

IL-1β is a proinflammatory cytokine implicated in metabolic inflammation and neuroinflammatory cascades [31,32,33,34,35]. In this study, brain IL-1 levels were significantly higher in the T2DM group compared with several comparator groups. The ALZ group also showed elevated IL-1, aligning with evidence supporting cytokine involvement in AD-related neuroinflammation [33,34,35]. CL-related changes in IL-1 should be interpreted cautiously; nevertheless, prior reports describing anti-inflammatory and antinociceptive properties of CL support further mechanistic evaluation in STZ-based models [36,37].

GLUT4 is an insulin-sensitive glucose transporter expressed in brain regions involved in glucose sensing and cognitive processes [38,39]. Brain GLUT4 levels were significantly higher in the ALZ + CL group compared to all other groups, and GLUT4 levels in the T2DM group were also higher than controls. Because we did not directly assess membrane translocation or upstream signaling pathways, the mechanism underlying these changes cannot be concluded from the present data. Future studies should investigate whether CL influences GLUT4 trafficking and related insulin-signaling nodes in the brain in these models [40,41].

In the present study, serum Aβ levels were higher in the ALZ group compared with control and T2DM groups, whereas brain Aβ levels were higher in the T2DM group than in the ALZ group. These tissue-specific patterns highlight that amyloid-related readouts may differ by model and compartment. Given that insulin resistance and hyperinsulinemia may impair Aβ clearance through competition for insulin-degrading enzymes [9,42,43,44,45], further work is needed to clarify how CL affects amyloid processing, clearance, and aggregation state in both T2DM and STZ-induced ALZ models.

Brain GLP-1 levels were higher in several groups compared to the control, including control + CL, T2DM-related groups, and ALZ + CL. Serum GLP-1 levels were highest in the T2DM + OA group, indicating a stronger effect of metformin than CL on circulating GLP-1 in this dataset. GLP-1 can influence central glucose metabolism and has been linked to cognitive outcomes in experimental and clinical contexts [46,47,48,49,50,51,52,53]; however, the direction and magnitude of GLP-1 changes may vary by model and assay, warranting targeted mechanistic studies in the current experimental settings.

AChE inhibitors are a recognized symptomatic approach in AD treatment [54]. In this study, CL was associated with reduced serum AChE in the ALZ group, while patterns in brain AChE differed by group and should be interpreted in the context of tissue-specific regulation. Prior work has reported that Turkish CL extracts, particularly leaf methanol extract, exhibit AChE and BuChE inhibitory activity alongside antioxidant effects [55]. Future studies should evaluate cholinergic outcomes alongside behavioral endpoints using standardized dosing and phytochemical profiling.

CL has been associated in previous studies with free radical scavenging capacity, modulation of inflammatory mediators, and cholinesterase-inhibitory activity [18,19]. The observed increase in GSH levels and reduction in serum Aβ and AChE in the present study may therefore be partly attributable to the antioxidant and polyphenol-rich profile of the fruit. Phenolic compounds, in particular, are known to influence redox balance and may indirectly modulate signaling pathways involved in neuroinflammation and metabolic regulation. Although the present study did not perform detailed phytochemical characterization, the biological effects observed are consistent with the known antioxidant and neuroactive properties of polyphenol-containing plant matrices. Future studies integrating quantitative phytochemical profiling and compound-specific analyses will be essential to identify the principal bioactive contributors.

The Morris Water Maze (MWM) test was used to assess hippocampal-dependent learning and spatial memory [14]. CL administration was associated with reduced escape latency/swimming time, particularly in the ALZ model group, consistent with improved task performance. As MWM outcomes can be sensitive to methodological variation, replication with standardized conditions and complementary behavioral measures would strengthen inference [56,57].

**Figure 5:**
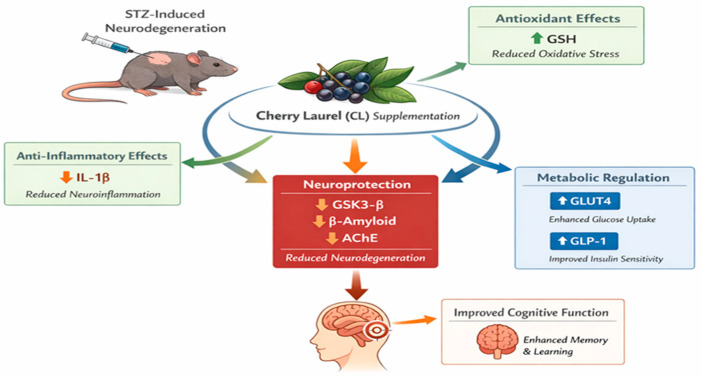
Proposed mechanistic model of cherry laurel (CL)-mediated neuroprotection in STZ-induced experimental models.

CL supplementation was associated with increased GSH levels and reduced IL-1β, suggesting attenuation of oxidative stress and neuroinflammation. Modulation of metabolic markers (GLUT4 and GLP-1) was observed alongside tissue-specific regulation of GSK3-β. In the Alzheimer model, reduced serum β-amyloid and acetylcholinesterase (AChE) levels were detected. These integrated biochemical changes were accompanied by improved performance in the Morris Water Maze test. Figure 5 represents a conceptual model based on measured parameters in the present study.

### 4.1. Strengths and Limitations

The main strength of this study is the use of two complementary experimental models (STZ-induced T2DM and nontransgenic sporadic AD) to investigate overlapping metabolic and neurodegenerative mechanisms within the same experimental framework. The simultaneous evaluation of behavioral outcomes (Morris Water Maze) together with serum and brain tissue biochemical markers provides an integrated assessment of cognitive and molecular changes. In addition, the tissue-specific analysis of biomarkers (serum vs. brain) enhances the mechanistic relevance of the findings.

Several limitations should be acknowledged. First, the study was conducted in experimental animal models, which may limit direct translational applicability to human disease. Second, mechanistic signaling pathways, including phosphorylation status and downstream intracellular mediators, were not directly assessed. In addition, the phytochemical composition of the administered cherry laurel preparation was not comprehensively characterized, and the exact daily intake of individual bioactive compounds was not quantified. A dose–response analysis was also not performed. Future studies incorporating detailed pathway investigations, quantitative phytochemical profiling, macronutrient-matched control designs, dose–response evaluation, and longer intervention periods would further clarify the mechanistic basis and translational relevance of cherry laurel supplementation.

### 4.2. Clinical Implications and Future Directions

The present findings suggest that CL fruit may have translational potential as a dietary adjunct targeting shared mechanisms between type 2 diabetes mellitus and sporadic Alzheimer’s disease, particularly oxidative stress, glucose dysregulation, and cholinergic imbalance. Although direct clinical extrapolation is premature, the observed improvements in cognitive performance and modulation of metabolic and inflammatory markers indicate that CL could be explored as a complementary strategy in metabolically driven cognitive impairment.

Future research should focus on (i) detailed mechanistic pathway analyses, including intracellular signaling and phosphorylation status, (ii) comprehensive phytochemical characterization to identify bioactive compounds responsible for the observed effects, (iii) dose–response and long-term safety studies, and (iv) well-designed clinical trials evaluating cognitive, metabolic, and inflammatory outcomes in at-risk or early-stage patient populations. Such studies will be essential to clarify the therapeutic relevance and translational applicability of CL in metabolic–neurodegenerative conditions.

### 4.3. Conclusions

Our findings suggest that CL supplementation is associated with improved glycemic control in the T2DM model and with changes in oxidative stress (GSH), inflammatory (IL-1), and amyloid/cholinergic-related measures (Aβ, AChE), alongside improved MWM performance in the STZ-induced nontransgenic ALZ model. Key limitations of this study include the reliance on STZ-induced models, assessments conducted at a sole dose and time point, and the lack of direct measurements regarding upstream signaling mechanisms such as pathway activation, transporter translocation, and amyloid aggregation states. Consequently, future studies should incorporate dose–response designs, detailed phytochemical characterization, and mechanistic assays to elucidate causal pathways and establish translational relevance.

## Figures and Tables

**Table 1:** Summary of experimental groups and interventions.

Group	Final n (Analysed)	Model/Induction	Treatment (Route)	Dose	Schedule/Duration
Control	5	Sham i.c.v. (aCSF)	None	—	aCSF 20 µL total/day i.c.v. on Days 1 and 3
Control + CL	5	Sham i.c.v. (aCSF)	*Cherry laurel* (gavage)	0.37 g/mL	aCSF i.c.v. Days 1 and 3 + CL daily for 21 days
T2DM	8	T2DM model: STZ i.p.	None	STZ 65 mg/kg	STZ i.p. on Day 1; diabetes defined as glucose > 200 mg/dL
T2DM + CL	8	T2DM model: STZ i.p.	*Cherry laurel* (gavage)	STZ 65 mg/kg; CL 0.37 g/mL	STZ Day 1 + CL daily for 21 days
T2DM + OA	7	T2DM model: STZ i.p.	Metformin (gavage)	STZ 65 mg/kg; Metformin 300 mg/kg/day	STZ Day 1 + metformin daily for 21 days
ALZ	9	Nontransgenic sporadic AD model: STZ i.c.v.	None	STZ 3 mg/kg	STZ i.c.v. on Days 1 and 3 (10 µL/ventricle; 20 µL/day total)
ALZ + CL	8	AD model: STZ i.c.v.	*Cherry laurel* (gavage)	STZ 3 mg/kg; CL 0.37 g/mL	STZ i.c.v. Days 1 and 3 + CL daily for 21 days

Footnote: Morris Water Maze (MWM) was performed for 5 days starting on Day 14. Animals were sacrificed on Day 22; serum and brain tissue biomarkers were measured.

**Table 2:** GSK3-β, GSH, IL-1, GLUT4, β-Amyloid, GLP-1 and AChE levels in brain tissues of the groups.

	Control (n:5)	Control + CL (n:5)	T2DM (n:8)	T2DM + CL (n8)	T2DM + OA (n:7)	ALZ (n:9)	ALZ + CL (n:8)	*p* Value *
**GSK3-β (pmol/L)**	37.27 ± 8.03 ^c^	25.13 ± 4.81	63.88 ± 15.75 ^a.f.g^	48.13 ± 19.81	43.45 ± 21.34	33.82 ± 21.9 ^c^	23.54 ± 10.67 ^c^	<0.001
**GSH (ng/L)**	68.48 ± 26.14 ^c.d.g^	115.25 ± 27.29 ^g^	157.1 ± 26.14 ^a.f^	145.59 ± 35.64 ^a.f^	121.09 ± 40.09 ^g^	89.21 ± 25.32 ^c.d.g^	168.76 ± 17.91 ^a.b.e.f^	<0.001
**IL-1 (ng/L)**	27.37 ± 9.32 ^c^	15.49 ± 2.49 ^c.d.e^	41.54 ± 5.45 ^a.b.f.g^	33.79 ± 12.04 ^b^	31 ± 6.55 ^b^	25.35 ± 4.05 ^c^	25.48 ± 1.75 ^c^	<0.001
**GLUT4 (µg/L)**	3.59 ± 0.54 ^d.g^	6.14 ± 1.43 ^g^	8.61 ± 3.03 ^g^	9.94 ± 5.02 ^a.g^	8.31 ± 2.41 ^g^	6.84 ± 2.19 ^g^	14.73 ± 2.13 ^a.b.c.d.e.f^	<0.001
**Aβ(µg/L)**	4.04 ± 0.57 ^b.c.d.e.f.g^	8.4 ± 0.86 ^a^	10.05 ± 1.95 ^a.f^	8.15 ± 2.02 ^a^	8.18 ± 2.33 ^a^	7.36 ± 1.5 ^a.c^	8.2 ± 1.41 ^a^	<0.001
**GLP-1 (pmol/L)**	0.4 ± 0.06 ^b.c.d.e.g^	0.83 ± 0.16 ^a^	1.13 ± 0.18 ^a.e.f^	0.92 ± 0.25 ^a.f^	0.82 ± 0.2 ^a.c^	0.65 ± 0.15 ^c.d.g^	0.91 ± 0.13 ^a.f^	<0.001
**AChE (nmol/L)**	89.19 ± 19.77 ^b.c^	48.86 ± 10.15 ^a.c.d.e.f.g^	127.63 ± 10.68 ^a.b.f.g^	115.27 ± 30.84 ^b.f.g^	100.67 ± 13.06 ^b^	98.59 ± 11.79 ^b.c.d^	86.05 ± 14.51 ^b.c.g^	<0.001

**GSK3-β:** Glycogen synthase kinase 3-beta; **GSH:** Glutathione; **IL-1:** Interleukin-1; **GLUT4:** Glucose transporter 4; **GLP-1:** Glucagon-like peptide-1; **AChE:** Acetylcholinesterase; **CL:** Cherry laurel; **T2DM:** Type 2 Diabetes Mellitus; **OA:** Oral antidiabetic; **ALZ:** Alzheimer. * One-way ANOVA test was used. Post hoc Tukey test was applied. There is a statistically significant difference compared to a: Control group; b: Control + CL group; c: T2DM group; d: T2DM + CL group; e: T2DM + OA group; f: Alzheimer group; g: Alzheimer + CL group.

**Table 3:** Serum GSK3-β, GSH, IL-1, GLUT4, β-Amyloid, GLP-1 and AChE levels of the groups.

	Control (n:5)	Control + CL (n:5)	T2DM (n:8)	T2DM + CL (n:8)	T2DM + OA (n:7)	ALZ (n:9)	ALZ + CL (n:8)	*p* Value *
**GSK3-β (pmol/L)**	51.97 ± 9.89	58.45 ± 12.17	56.54 ± 7.49	63.58 ± 7.49	57.74 ± 15.21	55.43 ± 13.53	55.12 ± 12.35	0.668
**GSH (ng/L)**	114.92 ± 28.47	148 ± 56.53	97.08 ± 49.61 ^f.g^	143.55 ± 27.2	120.37 ± 44.54	154.06 ± 11.58 ^c^	161.95 ± 24.29 ^c^	0.009
**IL-1 (ng/L)**	36.3 ± 4.68	34.62 ± 5.42 ^f^	40.91 ± 2. 44	37.24 ± 5.25	38.48 ± 5.52	42.3 ± 3.9 ^b^	42.01 ± 2.04	0.01
**GLUT4 (µg/L)**	4.44 ± 1.1	4.8 ± 1.58	5.12 ± 1.02	3.45 ± 0.97 ^g^	5.16 ± 1.56	4.05 ± 1.32 ^g^	6.52 ± 1.35 ^d.f^	0.001
**Aβ (µg/L)**	13.1 ± 3.74 ^f^	17.29 ± 6.61	16.6 ± 3.58 ^f^	17.45 ± 3.34	17.95 ± 1.22	22.06 ± 2.31 ^a.c^	18.16 ± 3.63	0.004
**GLP-1 (pmol/L)**	0.93 ± 0.09 ^e.g^	1 ± 0.13 ^g^	0.86 ± 0.06 ^e.f.g^	0.93 ± 0.16 ^e.g^	1.16 ± 0.16 ^a.c.d^	1.08 ± 0.06 ^c^	1.23 ± 0.12 ^a.b.c.d^	<0.001
**AChE (nmol/L)**	98.2 ± 16.3 ^f.g^	79.4 ± 19.43 ^e.f.g^	125.44 ± 18.59 ^d.f.g^	98.65 ± 12.43 ^c.e.f.g^	121.46 ± 10.8 ^b.d.f.g^	164.36 ± 19.27 ^a.b.c.d.e^	155.12 ± 16.92 ^a.b.c.d.e^	<0.001

**GSK3-β:** Glycogen synthase kinase 3-beta; **GSH:** Glutathione; **IL-1:** Interleukin-1; **GLUT4:** Glucose transporter 4; **GLP-1:** Glucagon-like peptide-1; **AChE:** Acetylcholinesterase; **CL:** Cherry laurel; **T2DM:** Type 2 Diabetes Mellitus; **OA:** Oral antidiabetic; **ALZ:** Alzheimer. * One-way ANOVA test was used. Post hoc Tukey test was applied. There is a statistically significant difference compared to a: Control group; b: Control + CL group; c: T2DM group; d: T2DM + CL group; e: T2DM + OA group; f: Alzheimer group; g: Alzheimer + CL group.

**Table 4:** Serum biochemical parameter levels of the groups.

	Control (n:5)	Control + CL (n:5)	T2DM (n:8)	T2DM + CL (n:8)	T2DM + OA (n:7)	ALZ (n:9)	ALZ + CL (n:8)	*p* Value *
**Urea (mg/dL)**	34.4 ± 3.05 ^c.d.e^	44.6 ± 2.51 ^c.d.e.g^	63.5 ± 9.53 ^a.b.f.g^	70.88 ± 5.03 ^a.b.e.f.g^	60.57 ± 2.37 ^a.b.d.f.g^	39.11 ± 4.26 ^c.d.e^	32.75 ± 4.43 ^b.c.d.e^	<0.001
**Creatinine (mg/dL)**	0.33 ± 0.01	0.45 ± 0.03	0.39 ± 0.04	0.35 ± 0.02	0.28 ± 0.04	4.28 ± 11.89	0.31 ± 0.03	0.631
**ALT (U/L)**	54.4 ± 3.36 ^c.d.e.g^	53 ± 3.16 ^c.d.e.g^	39.88 ± 4.91 ^a.b.f^	41.38 ± 4.07 ^a.b.f^	40.29 ± 5.65 ^a.b.f^	49 ± 6.2 ^c.d.e.g^	38.75 ± 3.49 ^a.b.f^	<0.001
**AST (U/L)**	40.8 ± 3.42	42.6 ± 2.3	45.88 ± 2.64 ^d^	38.63 ± 4.47 ^c.e^	42.14 ± 7.2 ^d^	45.89 ± 5.82	43.75 ± 2.92	0.029
**Cholesterol (mg/dL)**	63.6 ± 3.36 ^d.f^	59.6 ± 2.07 ^f^	67.63 ± 3.2 ^d.f.g^	57.88 ± 4.22 ^a.c.e.f^	64.29 ± 2.93 ^d.f.g^	83.25 ± 3.37 ^a.b.c.d.e.g^	61 ± 3.67 ^c.e.f^	<0.001
**TG (mg/dL)**	72.6 ± 18.72 ^b.c.d.g^	42.2 ± 2.77 ^a.e.f.g^	61.88 ± 3.94 ^a.d.g^	52 ± 3.21 ^a.c.e.f^	63.14 ± 3.48 ^b.d.g^	74.63 ± 3.25 ^b.d.g^	55.89 ± 4.26 ^a.b.c.e.f^	<0.001
**HDL (mg/dL)**	38 ± 1.58 ^e.g^	42.8 ± 2.59 ^c.g^	35.5 ± 2.62 ^b.d.e.f.g^	40.75 ± 4.27 ^c.g^	44.86 ± 3.44 ^a.c.g^	41.78 ± 3.15 ^c.g^	60.5 ± 2.45 ^a.b.c.d.e.f^	<0.001
**LDL (mg/dL)**	11.2 ± 1.92 ^b^	7 ± 2 ^a.c.d.e.f.g^	14.25 ± 2.25 ^b^	11.63 ± 1.77 ^b^	13.43 ± 2.51 ^b^	14.75 ± 2.66 ^b^	13.11 ± 2.03 ^b^	<0.001

**CL:** Cherry laurel; **T2DM:** Type 2 Diabetes Mellitus; **OA:** Oral antidiabetic; **ALZ:** Alzheimer; **ALT**: Alanine amino transferase; **AST:** Aspartate amino transferase; **TG:** Triglyseride; **HDL:** High-density lipoprotein; **LDL:** Low-density lipoprotein. * One-way ANOVA test was used. Post hoc Tukey test was applied. There is a statistically significant difference compared to a: Control group; b: Control + CL group; c: T2DM group; d: T2DM + CL group; e: T2DM + OA group; f: Alzheimer group; g: Alzheimer + CL group.

**Table 5:** Swimming times of animals (sec) varied with MWM.

	Control (n:5)	Control + CL (n:5)	T2DM (n:8)	T2DM + CL (n:8)	T2DM + OA (n:7)	ALZ (n:9)	ALZ + CL (n:8)	*p* Value *
1. day	46.01 (40.88–59.91)	37.96 (9.93–59.83)	44.36 (36.35–54.05)	45.855 (34.81–58.68)	51.87 (35.05–58.17) ^g^	44.35 (29.62–59.12)	31.985 (15.92–44.41) ^e^	0.023 ‡
2. day	57.46 (22.32–58.63)	9.2 (4.47–60)	43.65 (31.7–54.54)	36.915 (8.32–58.17)	43.45 (27.09–53.38)	22.77 (11.93–60)	18.275 (5.5–34.96)	0.013 ‡
3. day	29.85 (12.73–36.46)	13.12 (4.64–59.84)	36.185 (6.76–58.98)	45.02 (22.65–55.21) ^g^	28.86 (20.84–51.55)	17.87 (2.7–46.32)	11.465 (5.2–31.26) ^d^	0.007 ‡
4. day	10.32 (9.7–24.67)	7.13 (4.23–50.78)	32.03 (16.89–54.16) ^g^	23.155 (2.9–48.7)	27.79 (12.97–34.88)	20.9 (7.65–31.52)	6.345 (1.41–17.32) ^c^	0.005 ‡
5. day	4.1 (1.57–11.91)	9.11 (3.48–18.59)	17.49 (8.9–38)	12.095 (5.78–50.72)	14.6 (7.57–31.11)	20.55 (0.64–40.35)	13.43 (2.46–47.17)	0.133 ‡

**MWM:** Morris Water Maze; **CL:** Cherry laurel; **T2DM:** Type 2 Diabetes Mellitus; **OA:** Oral antidiabetic; **ALZ:** Alzheimer. ‡**: Kruskal–Wallis test was used.** * One-way ANOVA test was used. Post hoc Bonferroni correction was applied. There is a statistically significant difference compared to c: T2DM group; d: T2DM + CL group; e: T2DM + OA group; g: Alzheimer + CL group.

## Data Availability

The datasets used and/or analysed during the current study are available from the corresponding author on reasonable request.
